# An efficient protocol for inducing pseudopregnancy using estradiol dipropionate and follicular development associated with changes in reproductive hormones after prostaglandin F2alpha treatment in pseudopregnant sows

**DOI:** 10.1186/1477-7827-9-157

**Published:** 2011-12-14

**Authors:** Michiko Noguchi, Koji Yoshioka, Chie Suzuki, Seigo Itoh, Hiroyuki Kaneko

**Affiliations:** 1 Pathology and Pathophysiology Research Division, National Institute of Animal Health, Tsukuba, Ibaraki 305-0856, Japan; 2 Laboratory of Veterinary Internal Medicine, Azabu University, Sagamihara, Kanagawa 229-8501, Japan; 3 Animal Development and Differentiation Research Unit, National Institute of Agrobiological Sciences, Tsukuba, Ibaraki 305-8602, Japan

**Keywords:** estradiol dipropionate, estrus synchronization, follicular development, inhibin, PGF2alpha, pseudopregnancy

## Abstract

**Background:**

Utilization of estrus synchronization program in livestock industry would provide greater options for reproductive management in herd. To develop a convenient method for estrus synchronization in pigs, we determined the effective protocol using estradiol dipropionate (EDP) for the establishment of pseudopregnancy and investigated follicular development during the estrus synchronization with prostaglandin F2alpha (PGF2alpha) in association with reproductive hormone profiles in pseudopregnant sows.

**Methods:**

In Experiment 1, the effective dose (0, 10, 20, or 30 mg) and timing (5, 8, 11 or 13 days after ovulation) of a single administration of EDP in cyclic pigs for the induction of pseudopregnancy were investigated. In Experiment 2, four pseudopregnant sows were treated with PGF2alpha twice at a 24-h interval between 24 and 28 days after EDP treatment. The changes in plasma concentrations of reproductive hormones were analyzed by time-resolved fluoroimmunoassay. Follicular development and ovulation following PGF2alpha administration were monitored by transrectal ultrasonography.

**Results:**

High efficiency (greater than 80%) of pseudopregnancy was achieved with a single treatment with 20 mg of EDP at 8 and 11 days after ovulation (equivalent to 9-13 days after the onset of estrus). Plasma estradiol-17beta concentrations in pseudopregnant sows were significantly higher between 12 h and 7 days than before EDP treatment. Total inhibin concentrations significantly decreased following EDP treatment and remained low for 14 days. The number of small follicles was increased from 6.3 +/- 2.6 at PGF2alpha treatment to 22.8 +/- 4.8 at 3 days later; this was associated with increased plasma concentrations of inhibin. Onset of estrus was detectable in all sows on 5.3 +/- 0.3 days after PGF2alpha treatment and the number of ovulated follicles was 15.5 +/- 1.4 detected at 7.6 +/- 0.2 days after the treatment.

**Conclusions:**

This study has defined the effective dose and timing of EDP treatment for inducing pseudopregnancy in cyclic pigs. Our results also indicated that EDP caused a lowering of inhibin concentrations during pseudopregnancy and small numbers of follicles from 20 to 28 days after EDP. In contrast, EDP-induced pseudopregnancy appears to have no adverse effect on follicular development and subsequent ovulation following PGF2alpha administration.

## Background

In pigs, estrogen stimulation from the conceptus plays a role in maternal recognition of the conceptus for the establishment of pregnancy [1]. Repeated treatment with estradiol benzoate (EB) [2] or estradiol valerate (EV) [3] can extend the luteal function in cyclic pigs as a substitute for the signals from the conceptus, resulting in pseudopregnancy. Because pseudopregnant pigs exhibit estrus within a given period after prostaglandin F_2α _(PGF_2α_) administration [2-4], this protocol is applicable for estrus synchronization. However, the traditional procedure requires considerable effort and cost for inducing pseudopregnancy by multiple administrations of EB or EV.

Estradiol dipropionate (EDP), which is a human pharmaceutical, exhibited prolonged estradiol-17β effects compared with EB and EV in rats [5] and humans [6]. Our previous study showed that a single administration of EDP at 12 days after the onset of estrus could also induce pseudopregnancy in gilts and plasma estradiol-17β concentrations in pseudopregnant gilts were maintained at high levels for 9 days after EDP treatment [4]. However, the optimum dose and timing of EDP treatment to induce pseudopregnancy in pigs has not yet been determined.

Although EB delayed new follicular wave emergence after follicular aspiration in cattle [7], the effect of high concentrations of estradiol-17β on follicular development in pigs is not clear. The analysis of follicular dynamics during treatments is important for development of an effective estrus synchronization protocol. The developmental stage of follicles at the time of induced luteolysis with PGF_2α _affected the interval from treatment to ovulation in cattle [8]. In previous reports, 83% to 100% of pseudopregnant pigs induced by EB [2,9] or EDP [4] exhibited estrus after PGF_2α _treatment [2,4,9], while the interval from the treatment to estrus ranged from 4 to 7 days [2-4,9]. To explain this variation, morphometric analysis of not only changes in corpora lutea (CL), but also in follicular development before and after PGF_2α _treatment, was required.

Inhibin levels in the circulation are recognized to reflect the number of antral follicles present on ovaries in cows [10], goats [11], and pigs [12]. Since the profiles of inhibin A and total inhibin during the porcine estrous cycle clearly corresponded with changes in the number of follicles more than 3 mm in diameter detected by ultrasonography [12], measurement of inhibin concentrations in plasma may be useful for understanding the alteration of follicular dynamics in pseudopregnant pigs. Moreover, exogenous estrogen affects the kinetics of various reproductive hormones, such as suppression of gonadotropin-releasing hormone secretion in the hypothalamus [13] and decreases of follicle-stimulating hormone (FSH) and luteinizing hormone (LH) concentrations in peripheral blood [13-15]. Furthermore, secretory patterns of LH, prolactin and progesterone during pseudopregnancy do not imitate the implantation period of natural pregnancy in the pig [16]. Thus, comprehensive analysis of reproductive hormones, including inhibin, estradiol-17β, progesterone, LH, and FSH, in pseudopregnant pigs should be helpful to establish the porcine estrus synchronization protocol using EDP and PGF_2α_.

The aims of the present study were: (1) to determine the effective dose and timing of EDP administration in cyclic pigs for the induction of pseudopregnancy and the profiles of inhibin and other reproductive hormones in pseudopregnant pigs induced by EDP treatment, and (2) to investigate the relationship between the profiles of reproductive hormones and the changes in ovarian structures after PGF_2α _treatment in pseudopregnant sows.

## Methods

### Animals and treatments

All animal-related procedures employed in this study were approved by the Institutional Care and Use Committee for Laboratory Animals of the National Institute of Animal Health. This experiment was carried out at the National Institute of Animal Health in Tsukuba, Japan. Five mature cyclic gilts that exhibited more than nine estrous cycles after puberty (Landrace [L] × Large White [W]; 20.2 ± 1.1 [mean ± SEM] months, 180 ± 13.8 kg) and 35 sows (L, n = 6; W, n = 2; and LW, n = 27; 27.9 ± 2.9 months, 201.0 ± 5.1 kg, 2.4 ± 0.5 parities) were used. Estrus was checked twice daily using a mature boar by the method previously described [12]. All sows were in estrus within 10 days after weaning and exhibited one or more estrous cycles (averaged on 4.2 ± 0.5). At least one estrous cycle of normal length (18-24 days) was observed in each pig immediately before the start of the study.

### Treatments and experimental design

#### Experiment 1

In the first part of experiment 1, to determine the dosage of EDP (Ovahormone Depot; ASKA Pharmaceutical Co., Ltd., Tokyo, Japan) required to establish pseudopregnancy, animals were given 0 (vehicle of 20% [vol/vol] benzyl benzoate in sesame oil, n = 4), 10 (n = 4), 20 (n = 5), or 30 mg (n = 4) of EDP intramuscularly once at either 10-13 days after the onset of estrus. In the second part of experiment 1, sows were treated with 20 mg of EDP at 5 (n = 5), 8 (n = 5), 11 (n = 4), or 13 days (n = 5) after ovulation to examine precisely the effect of timing of EDP treatment on the induction of pseudopregnancy. Five, 8, 11 and 13 days after ovulation were equivalent to 6-7, 9-10, 12-13 and 14-16 days after the onset of estrus, respectively. Ovulation was detected using transrectal ultrasonography in all sows as previously described [17]. Animals were checked for estrus from 17 days after the onset of estrus until the end of the subsequent estrus was observed, or until 24 days after treatment if they did not exhibit a subsequent estrus. Each animal was fitted with an indwelling catheter in the auricular vein at least 3 days before each treatment. Blood samples were collected at least daily from the day of catheterization until 10 days after the onset of the subsequent estrus, or until 24 days after the treatment if estrus was not observed. Plasma was recovered after centrifugation of blood and stored at -20°C. Pseudopregnancy was defined as the absence of estrus with plasma progesterone concentrations of more than 5 ng/mL, maintained until 24 days after EDP treatment, as previously described [18,19].

#### Experiment 2

Four sows in which pseudopregnancy was induced by a single administration of 20 mg EDP at 9-13 days after the onset of estrus (see Results) were used to determine the changes in the ovaries in relation to circulating hormone profiles after luteolysis. Pseudopregnant sows were treated with PGF_2α _as 15 mg dinoprost (Panacelan Hi; Meiji Seika, Tokyo, Japan) intramuscularly twice at a 24-h interval 24-28 days after EDP treatment. Changes in ovarian structures, such as follicular growth, ovulation, and regression of CL were monitored daily with transrectal ultrasonography as previously described [12], starting 4 days before the first PGF_2α _treatment and ending 7 days after the subsequent ovulation. Briefly, ovaries were scanned with an ultrasound machine (SSD-900SE; Aloka, Tokyo, Japan) equipped with a 7.5-MHz linear array transducer. Follicles were identified as anechoic (echo-free) spherical structures and classified by size as small (≥ 3 and < 6 mm in diameter) or large (≥ 6 mm in diameter). Growth and regression of CL were expressed in terms of the mean diameter of the largest CL in each sow on each day. In addition, after sows had been deemed to be in estrus as defined during 6-h observations by their first standing response to an intact boar, the ovaries were scanned at 6-h intervals to detect the timing of ovulation.

Blood samples were collected every 12 h throughout the study via an indwelling catheter in the auricular vein and every 6 h from the first PGF_2α _treatment to the end of the subsequent estrus to characterize the hormonal profiles during the periovulatory period. Plasma was recovered after centrifugation of blood and stored at -20°C.

### Hormone assay

All plasma samples were measured by time-resolved fluoroimmunoassay (Tr-FIA) for total inhibin, estradiol-17β, progesterone, LH, and FH concentrations. Concentrations of total inhibin were determined by Tr-FIA as previously described [12]. The intra-assay and interassay coefficient of variations (CVs) were 10.7% and 15.8%, respectively. Plasma concentrations of estradiol-17β and progesterone were measured with a Tr-FIA kit (DELFIA Estradiol and Progesterone kits; PerkinElmer Japan, Yokohama, Japan), as previously reported [20]. The intra-assay and interassay CVs were 8.4% and 10.1% for estradiol-17β and 7.8% and 7.4% for progesterone, respectively. Plasma concentrations of LH and FSH were determined using Tr-FIA methods previously described by Noguchi et al. [20] and Ohnuma et al. [21], respectively. The intra-assay and interassay CVs were 6.8% and 8.4% for LH and 7.2% and 10.0% for FSH, respectively.

### Statistical analyses

The duration of the LH surge was considered to be the time from the onset to the end of the LH surge, as defined by a previously described method [22]. Briefly, the onset or the end of a surge was defined as the first or last LH sample that exceeded the presurge baseline by twice of standard deviation and did not return to that baseline within 6 h. Data pertaining to follicular growth and hormonal profiles were subjected to analysis of variance (ANOVA) for repeated measures [23]. When a significant effect was detected by ANOVA, the significance of the difference between means was determined by Tukey's test. Regression analysis between EDP dosage and the peak concentration of plasma estradiol-17β, was also performed. All data were analyzed using the GLM or REG procedure of SAS (SAS Institute, Inc., Cary, NC). A value of *P *< 0.05 indicated statistical significance. Treatment effects on the incidence of pseudopregnancy were analyzed using χ^2 ^analysis.

## Results

### Induction of pseudopregnancy with different dosages and timing of EDP administration

Efficiency of the induction of pseudopregnancy in each EDP treatment group is shown in Table 1. Of the different dosages of EDP administration, the proportion of pseudopregnant pigs treated with 20 mg was higher than the proportion of pigs that underwent no treatment (*P *< 0.05). There was a positive correlation between EDP dosages and peak plasma concentrations of estradiol-17β after treatment (r = 0.66, *P *< 0.05).

**Table 1:** Efficiency of the induction of pseudopregnancy with different dosages and timing of EDP treatment

Category	No. of pigs	No. of pseudopregnancies (%)
Dosage*		
0 mg	4	0 (0)^a^
10 mg	4	1 (25.0)^ab^
20 mg	5	4 (80.0)^b^
30 mg	4	3 (75.0)^ab^
Timing^†^		
Day 5	5	3 (60.0)
Day 8	5	4 (80.0)
Day 11	4	4 (100)
Day 13	5	2 (40.0)

*Pigs were treated with each dosage of EDP once at 10-13 days after the onset of estrus.

^†^Pigs were treated with 20 mg EDP once at 5, 8, 11 or 13 days after ovulation.

^a, b^Values with different superscripts within a column in the different dosage differ significantly (*P *< 0.05).

A high incidence of pseudopregnancy in pigs treated with EDP at 8 or 11 days after ovulation (equivalent to 9-13 days after the onset of estrus) was observed, while the incidence did not differ significantly with the timing of EDP treatment. Mean peak concentrations of estradiol-17β (130.2 to 309.0 pg/mL) did not differ significantly among groups. When EDP was administered at 13 days after ovulation (14-16 days after the onset of estrus), plasma progesterone concentrations from 12 h onwards after treatment were lower (*P *< 0.05) than those 2 days before treatment. In contrast, there was no significant change in plasma progesterone levels for 3 days before or after EDP treatment in the other groups (data not shown).

### Hormonal profiles during pseudopregnancy

**Figure 1:**
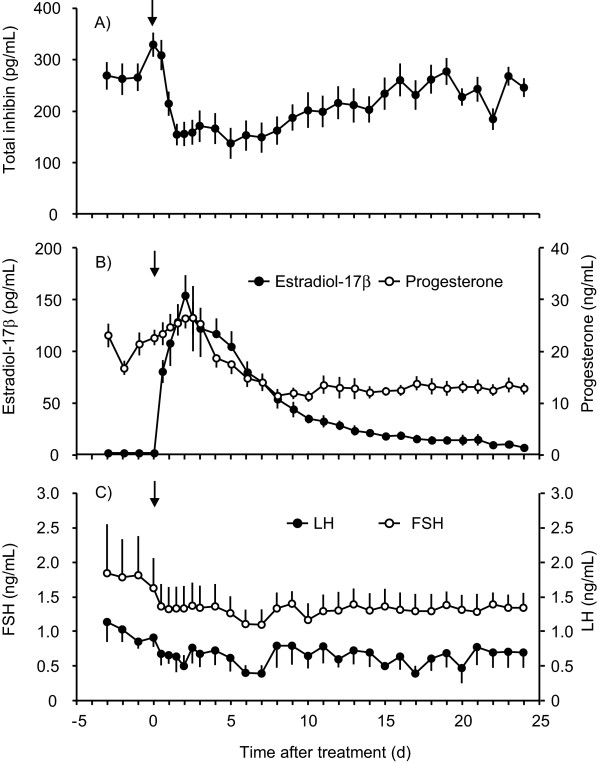
**Profiles of reproductive hormones in pseudopregnant sows**. Changes in the plasma concentrations of total inhibin (A), estradiol-17β and progesterone (B), and LH and FSH (C) in 10 pseudopregnant sows were indicated. Pseudopregnancy was induced by a single treatment with 20 mg EDP at 9-13 days after the onset of estrus. Values are means ± standard error of means (SEM).

The overall pseudopregnancy rate in pigs treated once with 20 mg of EDP at 9-13 days after the onset of estrus was 85.7% (12/14). Of these 12 pseudopregnant pigs, profiles of total inhibin, estradiol-17β, progesterone, LH, and FSH in 10 sows are shown in Figure 1. Total inhibin levels in plasma were reduced (*P *< 0.05) from 1 to 14 days compared with that on the day of EDP treatment, and were increased (*P *< 0.05) at 16, 18, 19, and 23 days compared with the minimum level at 5 days after EDP treatment (Figure 1A). Plasma estradiol-17β concentrations in pseudopregnant sows were higher (*P *< 0.05) from 0.5 to 7 days than those before EDP treatment (Figure 1B). Plasma progesterone levels in pseudopregnant sows were maintained at more than 5 ng/mL throughout the experimental period, while progesterone concentrations from 7 to 24 days were lower (*P *< 0.05) than on the day of EDP treatment (Figure 1B). While FSH levels did not change significantly during the study period, plasma LH concentrations were lower (*P *< 0.05) at 2, 6, 7, 15, 17, and 20 days than 3 days before EDP treatment (Figure 1C).

### Exhibition of estrus, ovarian changes, and hormonal profiles after PGF_2α _treatment in pseudopregnant pigs

**Figure 2:**
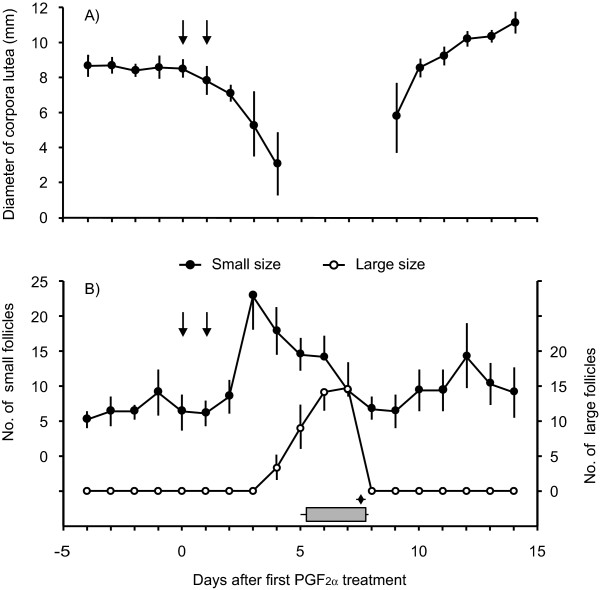
**Ovarian changes after PGF_2α _treatment in pseudopregnant sows**. Changes in the diameter of maximum-sized corpora lutea (A) and numbers of small and large follicles (B) after PGF_2α _treatment in pseudopregnant sows (n = 4) were indicated. Arrows in each panel indicate treatment with PGF_2α_. Estrus (shaded bar) and ovulation (rhombus) are shown at the bottom of figure. Values are means ± SEM.

The onset of estrus was detectable in all sows 5.5 ± 0.1 days after the first PGF_2α _treatment. The mean diameter of the largest CL was lower (*P *< 0.05) 4 days after the first PGF_2α _treatment than at treatment, and CL were not identified for the next 4 days (Figure 2A). Formation of CLs was identically observed at 2 days after ovulation (9 days after the PGF_2α _treatment) in all sows, and the CL diameter markedly increased thereafter. The mean number of small follicles (≥ 3 and < 6 mm in diameter) at 3 days after the PGF_2α _treatment was increased (*P *< 0.05) compared with the day of the treatment, and then decreased again (*P *< 0.05) by 8 days after the treatment (Figure 2B). Large follicles (≥ 6 mm in diameter) were not observed between 3 days before and 3 days after the treatment, but were present at 4 days after treatment (Figure 2B). The mean numbers of large follicles between 5 and 7 days after the treatment were greater (*P *< 0.05) than those at 3 days after the treatment. Ovulation was detected in all sows at 7.6 ± 0.2 days after PGF_2α _treatment, and the number of ovulated follicles was 15.5 ± 1.4.

**Figure 3:**
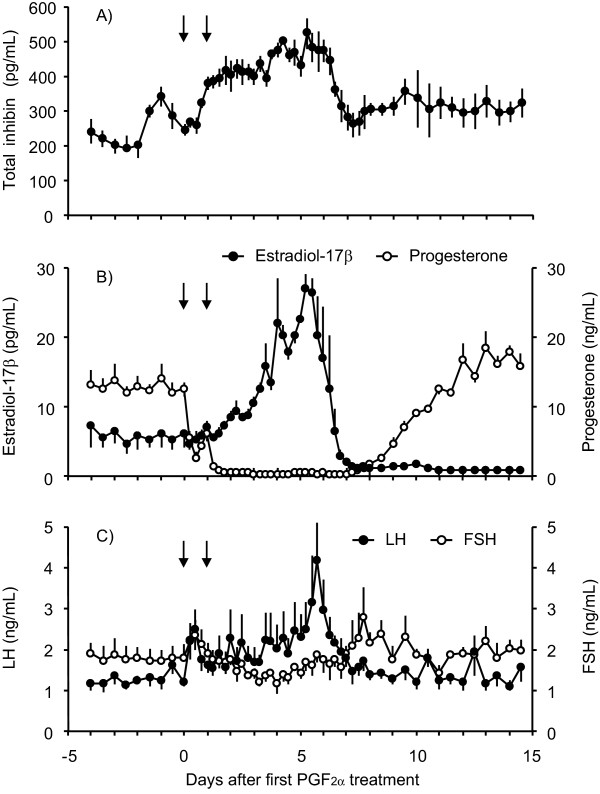
**Profiles of reproductive hormones after PGF_2α _treatment in pseudopregnant sows**. Changes in the plasma concentrations of total inhibin (A), estradiol-17β and progesterone (B), and LH and FSH (C) after PGF_2α _treatment in pseudopregnant sows (n = 4) were indicated. Arrows in each panel indicate treatment with PGF_2α_. Values are means ± SEM.

The mean concentration of total inhibin was higher 4 days after PGF_2α _treatment than that at the treatment (461.7 ± 10.8 pg/mL vs. 215.8 ± 15.8 pg/mL, *P *< 0.05), and remained higher than 500 pg/mL for the next 2 days (Figure 3A). Total inhibin levels at 7 days after the treatment were lower than on the previous day (314.0 ± 45.7 pg/mL vs. 522.7 ± 45.0 pg/mL, *P *< 0.05), after which they did not change significantly. Concentrations of estradiol-17β were greater (*P *< 0.05) at 4 to 6 days after PGF_2α _treatment than before the treatment. At 7 days after treatment, estradiol-17β concentrations had decreased to 1.5 ± 0.2 pg/mL from the maximum value (27.1 ± 2.1 pg/mL) at 5 days after treatment (Figure 3B). Plasma progesterone levels decreased markedly (*P *< 0.05) from 6 h after PGF_2α _treatment and were at their minimum between 1 and 8 days after treatment (Figure 3B). An LH surge after PGF_2α _treatment was observed in three of four sows, peaking at 6.1 ± 0.2 days after the treatment (Figure 3C). Concentrations of LH and FSH increased (*P *< 0.05) at 6 and 8 days after the treatment, respectively, compared with those on the day of the treatment (Figure 3C).

## Discussion

The present study indicated that a single treatment with 20 mg of EDP at 9-13 days after the onset of estrus could induce pseudopregnancy with high efficiency of 80% or more in pigs. This study also revealed that administration of EDP not only prevented luteolysis in pigs but also influenced inhibin secretion. However, treatment with PGF_2α _in EDP-induced pseudopregnant pigs appeared to have no detrimental effect on final follicular development and ovulation.

High efficiency of pseudopregnancy (75-80%) was achieved in pigs treated once with 20 or 30 mg of EDP at 10-13 days after the onset of estrus, whereas only 25% of pigs treated with 10 mg of EDP became pseudopregnant. These results corresponded with those of a previous report [24] in which a single injection of 25 to 100 mg of slow-releasing estradiol-17β (SRE) incorporated into poly (D, L-lactide) microspheres, which was an experimental pharmaceutical preparation, induced pseudopregnancy in 86% to 88% of pigs, whereas the pseudopregnancy rate after treatment with 12.5 mg of SRE was 50%. Administration of a total dose of 20 to 50 mg of EB [2,25] or EV [3] in repeated administrations between 11 and 15 days of the porcine estrous cycle has also provided high efficiency of pseudopregnancy.

In the present study, pigs treated with EDP at 8 or 11 days after ovulation (9-13 days after the onset of estrus) became pseudopregnant with high efficiency (80-100%), but they did not differ significantly from those treated at 5 or 13 days after ovulation (40-60%). In pregnant pigs, concentrations of estrogen in the uterine lumen increase biphasically from 11 to 12 days and after 14 days of gestation, resulting in the first and second periods of maternal recognition [1]. The increase of estrogen concentrations *in vivo*, especially in the uterus from 11 to 12 days and 14 days of the estrus cycle, is also important for complete establishment of pseudopregnancy in cyclic pigs [2,25,26]. Plasma estradiol-17β concentrations in pigs administered EDP at 8 or 11 days after ovulation remained high for 6-8 days (data not shown). The variation in pseudopregnancy rates among the different timing of EDP administration may be influenced by estrogen levels in the uterus from 11 to 12 days and after 14 days of the estrous cycle, which correspond to the levels in plasma.

Our study indicated that plasma inhibin concentrations in pseudopregnant pigs were suppressed from 1 to 14 days after EDP administration. Exogenous estradiol induces follicular atresia in monkeys [14]. Expression of mRNA encoding inhibin α and β_A _subunits in atretic follicles was clearly decreased compared with those in healthy follicles in pigs [27]. Thus, it is suggested that high estradiol-17β concentrations in the plasma of EDP-treated pigs induce early follicular atresia, resulting in decreased plasma inhibin concentrations.

In our ultrasonographic examinations, the mean number of small follicles (≥ 3 and < 6 mm in diameter) detected on the day of PGF_2α _treatment, which ranged from 24 to 28 days after EDP treatment, was 6.3. This number was less than half of the number of the same-sized follicles (15.3) during the luteal phase (8 day before ovulation) of a normal estrous cycle [12]. In general, peripheral LH and FSH levels influence follicular emergence [28], and high concentrations of estradiol-17β cause suppression of the peripheral concentrations of LH in ovariectomized [29], cyclic [15], and pseudopregnant pigs [30]. In the present study, peripheral LH levels in pseudopregnant pigs were reduced at some points after EDP treatment, while an effect of EDP on FSH secretion was not evident in this study. The reduced number of follicles ≥ 3 mm in diameter 3 to 4 weeks after EDP treatment may have been caused by delayed follicular emergence in association with suppression of LH secretion.

Although inhibin secretion was initially suppressed following EDP treatment (approximate range, 100-200 pg/mL), it appeared to return to normal levels (approximate range, 200-300 pg/mL) 3-4 weeks later, corresponding to the baseline levels of the estrous cycle in the previous report [12]. Thereafter, changes in the number of follicles ≥ 3 mm in diameter and hormonal profiles in pseudopregnant sows from 3 days after PGF_2α _treatment were similar to those from the late luteal to the follicular phase of the estrous cycle in sows [12]. Moreover, the characteristics of estrus, preovulatory LH surge and ovulation in the PGF_2α_-treated pseudopregnant sows were also similar to those in cyclic sows [12,20,31-33] and pseudopregnant gilts induced by EB [34]. Thus, it is likely that the estrus synchronization protocol using EDP and PGF_2α _has no adverse effect on follicular development (from 3 mm in diameter) to ovulation.

## Conclusions

This study has defined the effective dose and timing of EDP treatment for inducing pseudopregnancy in cyclic pigs. Since EDP caused a lowering of inhibin concentrations during pseudopregnancy and small numbers of follicles from 20 to 28 days after EDP, administration of EDP may affect not only the maintenance of luteal function, but also follicular atresia and recruitment in the ovary. However, final follicular development and ovulation following PGF_2α _administration in the pseudopregnant pigs appear to be similar to those occurring during the natural estrous cycle. Thus, the use of EDP in combination with PGF_2α _should simplify and effective the porcine estrus synchronization protocol for not only gilts but also sows that had failed to conceive at the first estrus after weaning.

## Competing interests

The authors declare that they have no competing interests.

## Authors' contributions

This study represents the doctoral thesis of MN. MN performed the study and wrote the manuscript. KY made substantial contributions to the conception and the design of the study and participated in the data collection. KY also revised the manuscript critically and gave final approval of the version to be published. CS participated in the data collection and helped to draft the manuscript. SI revised the manuscript critically. HK participated in the Tr-FIA and critical discussion and revised the manuscript. All authors read and approved the final manuscript.
